# Extracorporeal Membrane Oxygenation in Burn Patients With Severe Inhalation Injuries: A Case Report

**DOI:** 10.7759/cureus.93771

**Published:** 2025-10-03

**Authors:** Namratha Mohan, Suyash Jain, Jad Zeitouni, Jinesh Lachmansingh, Alan Pang

**Affiliations:** 1 Department of Surgery, Texas Tech University Health Sciences Center, Lubbock, USA; 2 Department of Anesthesiology, Texas Tech University Health Sciences Center, Lubbock, USA

**Keywords:** acute respiratory distress syndrome (ards), burn trauma, extracorporeal membrane oxygenation support, inhalation lung injury, refractory hypoxemia

## Abstract

High-grade inhalational injuries are challenging to manage and increase the complexity and risk of respiratory dysfunction in burn patients. Treatment of these injuries typically requires aggressive supportive care, but in severe cases, conventional therapies do not provide adequate respiratory support. Extracorporeal membrane oxygenation (ECMO) has been increasingly utilized in clinical scenarios where patients require respiratory support; however, it has been used sparingly in burn patients. We present the case of a 52-year-old female patient with grade 4 inhalational injury who developed severe refractory hypoxemia and subsequent circuit clot formation, successfully detected and managed with venovenous ECMO (VV-ECMO). During ECMO support, circuit clot formation occurred but was promptly detected and effectively managed, highlighting both the utility of VV-ECMO in high-grade inhalational injuries with refractory hypoxemia and the importance of vigilant circuit monitoring.

## Introduction

Inhalational injuries are a broad classification of diseases that describe damage to the respiratory tract or lung tissue from inhaled heat, smoke, or chemical irritants [1]. The severity of inhalational injuries is determined via a grading system from 0 to 4, with grade 4 signifying the most severe [2]. The estimated incidence of inhalational injury in burn trauma patients has been shown to be as high as 39%, with more severe inhalational injury corresponding to increasingly severe burns [3]. Major causes of inhalational injuries include exposure to smoke from structural or wildland fires, industrial accidents, and explosions, where superheated air, particulate matter, and toxic gases such as carbon monoxide and hydrogen cyanide cause both thermal and chemical injury. Enclosed or poorly ventilated environments further amplify exposure, leading to airway damage, chemical pneumonitis, and severe hypoxemia that may progress to respiratory failure. The treatment for inhalational injuries, regardless of the stage, is supportive; supplemental humidified oxygen, airway suctioning, chest physiotherapy, early mobilization, aerosolized heparin, N-acetyl cystine, and bronchoscopy are all considered standard supportive care for inhalational injury in burn trauma patients [4].

Patients with severe inhalational injuries have been shown to have increasing incidences of pneumonia and acute respiratory distress syndrome (ARDS). ARDS is an acute disorder that typically presents 48-72 hours after a pulmonary or extrapulmonary insult and can progress to bilateral lung infiltration and increasing hypoxia [5]. Traditionally, the management of patients with ARDS is largely supportive and includes treatments such as improving oxygenation with positive end-expiratory pressure and conservative fluid management [6]. Patients with grade 4 inhalational injuries complicated by ARDS can develop refractory hypoxemia where conventional supportive therapy is inadequate and requires additional treatment.

Extracorporeal membrane oxygenation (ECMO) is an artificial circuit designed to bypass the lungs via drainage of the blood from the body, circulating it in a machine designed to oxygenate the blood and subsequently returning the blood to the body [7]. There are currently two major types of ECMO utilized in clinical practice: venoarterial (VA) and venovenous (VV). VA ECMO provides both pulmonary and hemodynamic stability by pumping oxygenated blood through the arterial system. In contrast, VV ECMO provides pulmonary support only by returning blood to the venous system. Thus, patients rely upon their own cardiac system for hemodynamic stability. A common cannulation strategy involves the use of a bichannel catheter (e.g., the Avalon catheter) and making a singular incision at the right internal jugular vein [7]. However, regardless of whether a physician is conducting VV or VA ECMO, initiation of anticoagulation prophylaxis is a crucial step.

Possible indications for ECMO include cardiac shock, cardiac arrest, and graft loss following a heart transplant. Pulmonary indications for VV ECMO include severe ARDS, refractory hypoxemia, or respiratory failure secondary to inhalational injury when conventional therapies are insufficient. Many studies have shown excellent survival-to-discharge rates, demonstrating the effectiveness of ECMO in providing adequate support with arteriovenous cannulation in patients who have rejected a recent heart transplant [8].

Three components are modifiable in ECMO management: blood/pump flow, sweep fraction of inspired oxygen (FiO_2_), and sweep flow. Blood and pump flow refer to the velocity of blood moving through the ECMO machine; this flow typically remains constant at 4-6 L/minute and can be adjusted by modifying the pump's revolutions per minute. Pump flow influences the oxygenation rate of the blood. Sweep refers to the gas that the blood interfaces with the oxygenation membrane. Sweep FiO_2_ is the oxygen concentration in sweep gas, and the flow is the velocity of the gas as it passes across the blood in the membrane. The normal sweep FiO_2_ is 1.0, and the normal sweep flow rate is 7-10 L/minute. Adjusting the sweep flow simultaneously influences the clearance of CO_2 _[8].

The use of ECMO in burn patients presents several challenges and considerations, including increased infection risk, wound management, fluid management, metabolic derangements, and increased bleeding risk [9]. With respect to increased bleeding risk specifically, ECMO-induced clots present a significant challenge, as patients need to be anticoagulated for the prevention of ECMO-induced clots. In patients with extensive burns, excision is often necessary for treatment; however, performing excision in those supported with VV ECMO poses unique challenges, as the systemic anticoagulation required to maintain circuit patency must be carefully balanced against the heightened bleeding risks from excision surgeries, fragile wound beds, and underlying coagulopathy.

By providing the necessary pulmonary support, the use of VV ECMO has the potential to help decrease recovery times following severe ARDS. Currently, there is an absence of literature discussing the effectiveness of utilizing ECMO in burn trauma patients who have severe inhalational injuries [10-12]. ECMO is a treatment modality that should be further explored in these patient populations.

This case was presented as an oral podium presentation at both the North American Burn Society, Mammoth Mountain, CA, on March 23, 2023; the Southern Burn Conference, Winston-Salem, NC, on October 10, 2023; and the Texas Society of Anesthesiologists’ Conference, San Antonio, TX, on September 7, 2024.

## Case presentation

**Figure 1:**
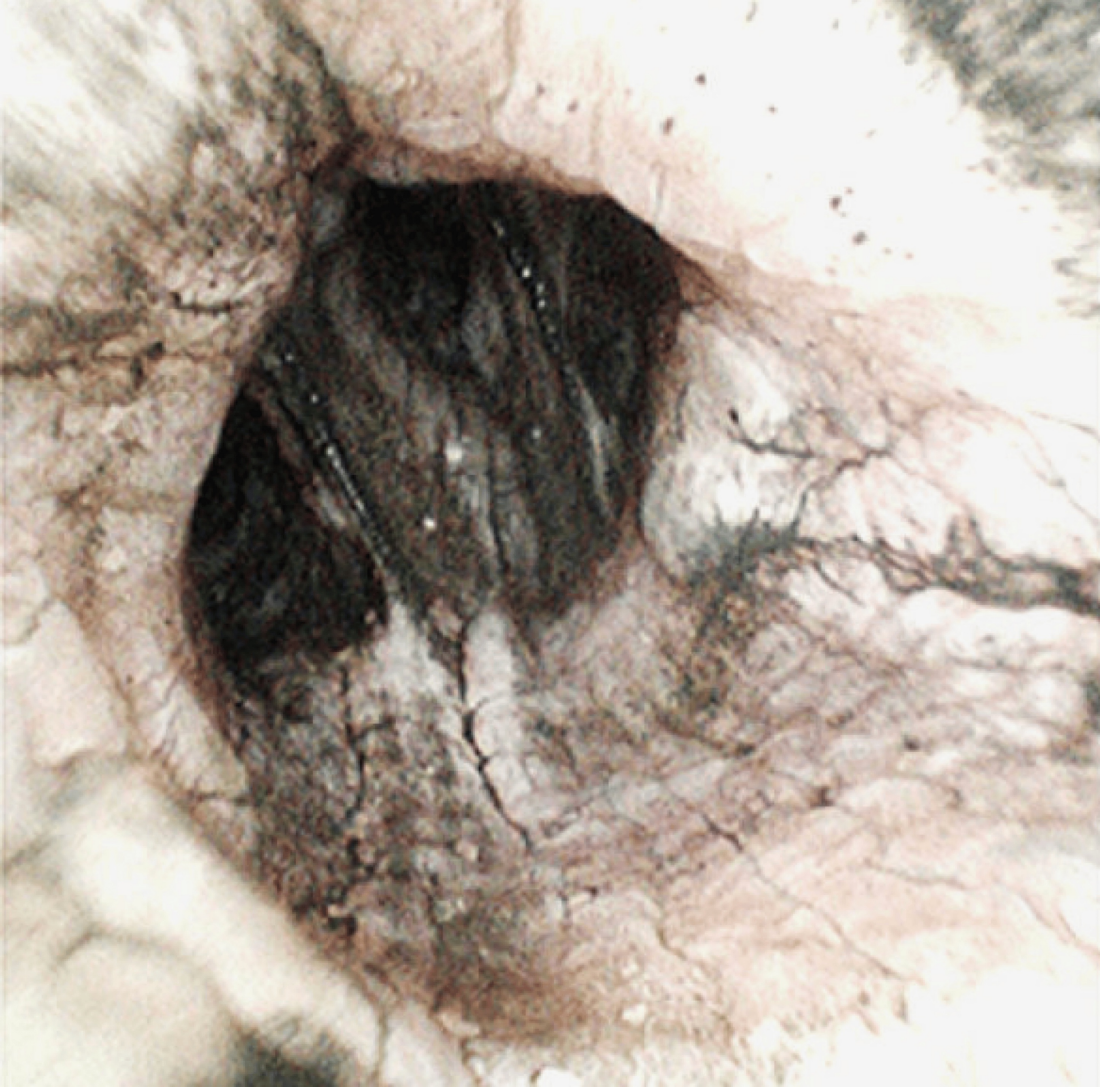
Bronchoscopy day 0 Diffuse carbonaceous deposits, mucosal sloughing, and airway narrowing consistent with grade 4 inhalational injury are seen throughout the main and terminal bronchi

**Figure 2:**
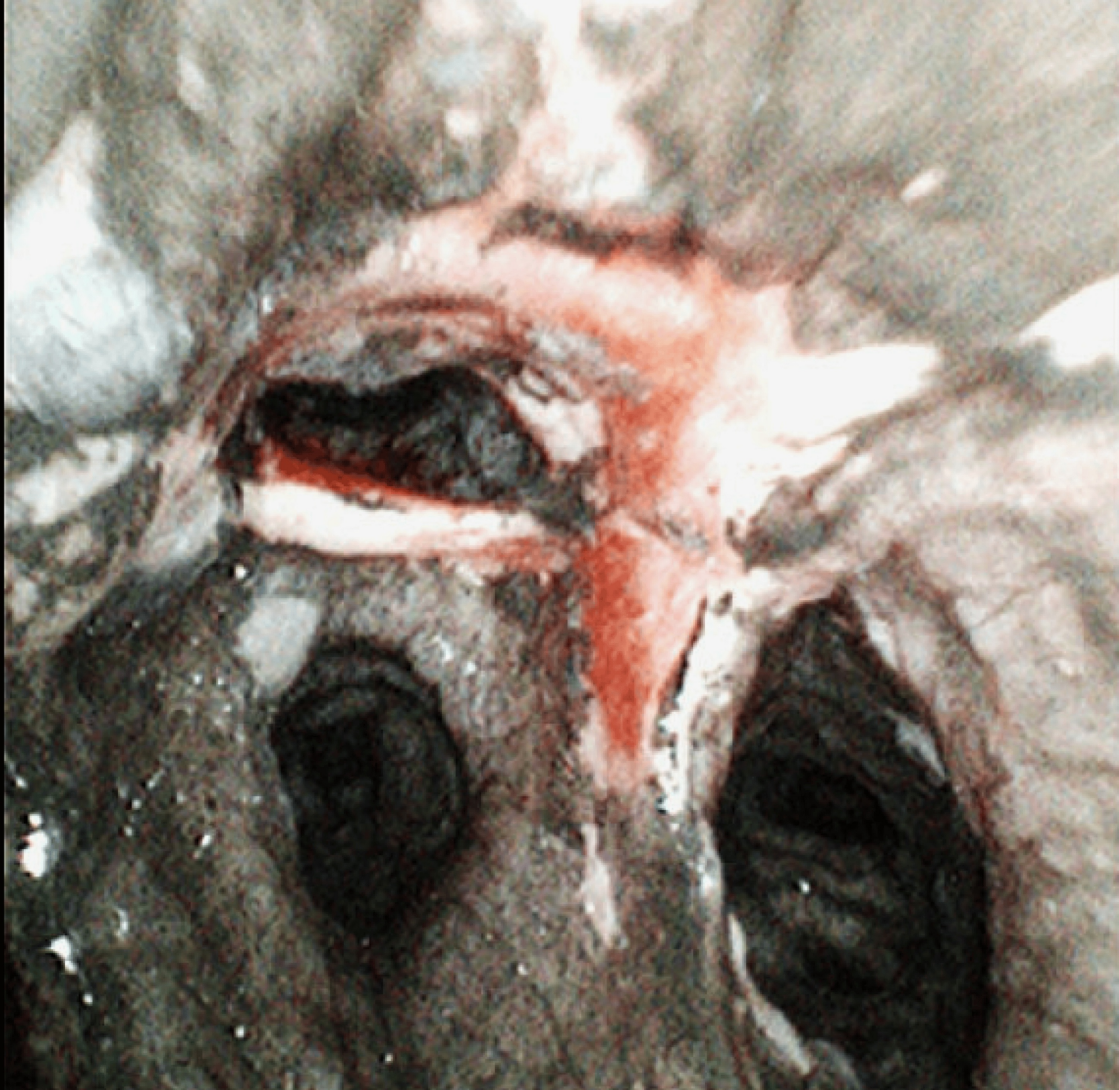
Bronchoscopy day 0 Severe mucosal erythema and necrosis with extensive soot deposition involving the carina and bilateral mainstem bronchi, indicative of extensive grade 4 inhalational injury

This case involves a 52-year-old female patient who was transferred from another facility following a structural fire incident at her home. Her total body surface area was 9.5% of her upper extremities, back, and face. She was intubated at the previous sending facility due to shortness of breath, and upon arrival at the current facility, bronchoscopy was performed, which revealed thick carbonaceous deposits throughout the main and terminal bronchi, a grade 4 inhalational injury, and mucosal sloughing (Figures 1, 2).

Her external thermal injuries were noted to be insignificant in comparison to the extent of her inhalation injuries. On the fourth day of the patient's hospital stay in the ICU, she was found to have worsening respiratory symptoms. She was previously on airway pressure release ventilation with an oxygen saturation of 82% (FiO_2_ 30%) and PaO_2_ of 40 mmHg (Pa/FiO_2_ ratio of 133.3 mmHg), and then was switched to high-frequency oscillating ventilation with no improvement in oxygen saturation in an attempt to serve as bridge therapy to improve oxygenation while limiting additional airway trauma from conventional ventilation pressures. At this time, the decision to utilize VV ECMO was made (4 L/minute flow, FiO_2_ 100%, sweep speed 2.5 L/minute). The patient's bilateral femoral veins were cannulated in the operating room.

For the next 11 days, the patient was sedated, paralyzed, and on pressor support; she also underwent serial bronchoscopy procedures to monitor the inhalational injury. Notable improvements were observed over time, and the patient tolerated ECMO well, despite a few complications. Attempts to reduce ECMO FiO_2_ to 90% were not tolerated on day 10 of hospitalization. She was weaned off pressor support slowly and received no pressors by day 13. Prone positioning began 16 hours a day beginning on day 14. A clot developed inside the machine circuit on day 15, and a therapeutic heparin drip (18 mg/kg/hour) was used. She tolerated a decrease in ECMO FiO_2_ to 50% with good partial pressure of oxygen saturation (93%) on day 16.

On day 17, the patient received VV ECMO. After decannulation, she was put back on the ventilator with a continued daily prone positioning regimen. By day 29, a tracheostomy was performed, and she was able to tolerate spontaneous ventilator settings. On day 33, the patient was transferred to a subacute facility on a tracheostomy collar with supplemental oxygen. To better illustrate the progression of illness, interventions, and response to treatment, a timeline of the patient's clinical course is provided in Table 1.

**Table 1:** Clinical course timeline Summary of key interventions and milestones during the patient's hospitalization for severe inhalation injury, including initiation of VV ECMO, prone positioning, and eventual recovery and transfer to a subacute facility TBSA: total body surface area; VV ECMO: venovenous extracorporeal membrane oxygenation; FiO_2_: fraction of inspired oxygen; pO_2_: partial pressure of oxygen

Day 0	Day 4	Days 5-15	Days 16-33
Presented with TBSA of 9.5% to the upper extremities, back, and face	Airway pressure release ventilation switched to high-frequency oscillating ventilation; no improvement in oxygen saturation noted	Patient sedated, paralyzed, and on pressor support	Tolerated decrease in ECMO FiO_2_ to 50% with good pO_2_ saturation (93%) on day 16
Bronchoscopy found carbonaceous deposits in the main and terminal bronchus, grade 4 inhalational injury, and mucosal sloughing	VV ECMO use begun; 4 L/minute flow, FiO_2_ 100%, sweep speed 2.5 L/minute	Tolerated ECMO well overall, besides an attempt to reduce ECMO FiO_2_ to 90% on day 10	Taken off VV ECMO on day 17 and put back on ventilator with daily prone positioning regimen after decannulation
		Weaned off pressor support slowly, receiving no pressors by day 13	Tracheostomy performed by day 29, patient able to tolerate spontaneous ventilator settings
		Prone positioning began for 16 hours daily, beginning on day 14	Patient transferred to subacute facility on tracheostomy collar with supplemental oxygen on day 33
		A clot formed inside the machine circuit on day 15 (despite a therapeutic heparin drip)	

## Discussion

Despite the fact that patients with burns and concomitant inhalational injury have a mortality of approximately 30%, the consensus for the standard of care when dealing with a patient with inhalational injuries is largely supportive [13]. Mechanical ventilation has been shown to be effective in treating lower grade inhalational injuries by providing positive airway pressure and using a lung-protective strategy. This strategy helps provide the oxygen needed by the patient while reducing the workload on the patient’s pulmonary system. A lung protective strategy helps reduce lung injury from overdistention by using lower pressures and tidal volumes [13].

With higher grade inhalational injuries, there is even more severe cellular damage resulting in the release of proinflammatory cytokines, increased mucosal sloughing with reduced clearance, weakened alveolar macrophages, and tissue necrosis [13]. Inhalational injuries that have been described as moderate to severe have shown that mechanical ventilation may not be enough to maintain adequate oxygen levels for optimal healing, thus reflecting the consideration of additional supportive therapies, such as ECMO, in the treatment of high-grade inhalational injuries complicated by refractory hypoxemia [14]. High-grade inhalational injuries (e.g., grade 3-4) often lead to severe airway edema and obstruction, diffuse alveolar damage and surfactant loss, and refractory hypoxemia and/or hypercapnia.

When the implementation of VV ECMO is considered, there are factors that need to be addressed; increased contact of blood with nonbiological surfaces significantly increases the chance of clot formation. This is due to several complex reactions that result in the induction of the coagulation cascade once venous blood encounters foreign material [15-17]. The introduction of a hypercoagulable state is an indication for anticoagulation protocols, and the need to administer anticoagulation is complicated by its contraindication in those with severe thermal injuries owing to the need for surgical intervention.

Moreover, VV ECMO use alone may not be as effective as a combination of treatments. For example, in this case study, physicians used prone positioning in addition to VV ECMO to help increase oxygenation levels. However, additional adjunctive therapies described in the literature include corticosteroid therapy to prevent tracheobronchial stenosis and the use of a protective ventilation strategy with low tidal volumes. Owing to the expansive range of patient responses to inhalational injuries and the highly subjective nature of grading, providing broad recommendations remains difficult.

This case specifically underscores the challenges of managing high-grade inhalational injuries, including the risk of respiratory failure and complications such as clot formation within the ECMO circuit, despite appropriate anticoagulation. The successful weaning of patients off ECMO after notable pulmonary recovery suggests that VV ECMO can serve as a critical bridge to recovery in situations where conventional ventilation strategies are insufficient.

## Conclusions

The success of this case highlights a novel indication for ECMO that could assist others with high-grade inhalational injuries and refractory hypoxemia and serve as a life-saving intervention when conventional therapies fail to provide adequate support. VV ECMO helps reduce the time needed for proper lung healing by offloading the work of the respiratory system to an artificial circuit. While this case demonstrates the feasibility of ECMO in burn patients, it also raises important considerations, including the risks associated with anticoagulation and the need for careful monitoring of potential complications, such as ECMO circuit clot formation. In general, ECMO has the potential to be another rescue strategy commonly utilized in managing severe inhalational injuries. Further direction in protocol recommendation requires gathering additional information on the impact of using ECMO, specific indications for its use, and controlled trials to determine its efficacy against standard protocols. Given the results of this case study, we recommend the consideration of VV ECMO as a supportive therapy in patients with high-grade inhalational injuries complicated by refractory hypoxemia.
